# Patients with *TP53*-Mutated Acute Myeloid Leukemia Receiving Intensive Induction Therapy Have Superior Outcomes Due to a Higher Rate of Salvage Therapy: A Single Institution, Retrospective Study

**DOI:** 10.3390/cancers16162784

**Published:** 2024-08-07

**Authors:** Nuttavut Sumransub, Gabriel K. Steinwand, Keith Cordner, Yoonkyu Lee, Qing Cao, Jeremy Allred, Veronika Bachanova, Mark Juckett, Craig Eckfeldt, Joseph E. Maakaron, Sean I. Tracy, Vidhyalakshmi Ramesh, Andrew C. Nelson, Sophia Yohe, Zohar Sachs

**Affiliations:** 1 Division of Hematology, Oncology and Transplantation, Department of Medicine, University of Minnesota, Minneapolis, MN 55455, USA; 2 M Health Fairview, Department of Pharmacy, University of Minnesota Medical Center, Minneapolis, MN 55455, USA; 3 Department of Medicine, University of Minnesota, Minneapolis, MN 55455, USA; 4 Bioinformatics and Computational Biology Graduate Program, University of Minnesota, Minneapolis, MN 55455, USA; 5 Biostatistics and Informatics, Clinical and Translational Science Institute, University of Minnesota, Minneapolis, MN 55455, USA; 6 Masonic Cancer Center, University of Minnesota, Minneapolis, MN 55455, USA; 7 Department of Laboratory Medicine & Pathology, University of Minnesota, Minneapolis, MN 55455, USA

**Keywords:** TP53 mutation, acute myeloid leukemia, salvage therapy

## Abstract

**Simple Summary:**

Mutations in the gene, *TP53*, define the most treatment-resistant subtype of acute myeloid leukemia (AML). Patients with *TP53*-mutated AML invariably develop disease recurrence after treatment, leading to short survival times. Previous studies have shown that less than 10% of these patients survive beyond 2 years after diagnosis. Moreover, the best treatment approach for these patients is still unclear. Our study is the first to analyze therapy results beyond first-line treatment, highlighting the importance of therapy sequencing in managing these patients. We found that sequential treatment with intensive chemotherapy followed by a less-intensive regimen achieved the highest remission rate of over 65%. Eligible patients should then receive blood stem cell transplantation from a donor, which significantly improves long-term survival. Our study provides crucial data to guide management and optimize therapy sequencing for better survival in this treatment-resistant disease.

**Abstract:**

Background: *TP53* mutations (*TP53*m) define the most treatment-refractory acute myeloid leukemia (AML) subtype. Optimal treatment approaches have not been established in this setting. We reviewed our institutional experience to identify therapy sequencing, treatment response, and survival patterns in these patients. Methods: This study was a single-center, retrospective cohort analysis. Results: Our cohort includes 86 *TP53*m and 337 *TP53* wild-type (*TP53*wt) adult AML patients. *TP53*m AML patients presented with lower bone marrow and peripheral blasts; none presented with hyperleukocytosis. Patients who received intensive treatment up front demonstrated superior overall survival (OS) over those receiving first-line non-intensive therapy (2-year OS 22% versus 7%; *p* = 0.02). However, the complete remission (CR) rates among the first-line intensive and non-intensive therapy groups were comparable (21.9% and 29.4%, respectively, *p* = 0.49). The improved OS is therefore attributed to superior cumulative CR in the intensive group. First-line intensively treated patients were more likely to receive and respond to salvage, leading to a cumulative CR rate of 65.7% (versus 29.4%, *p* = 0.003). Achieving CR at any point is strongly associated with superior survival outcomes with 2-year OS of 31% versus 0% for those not achieving CR ever (*p* < 0.01). Conclusions: We find that *TP53*m AML rarely presents with oncological emergencies, suggesting that clinical trial enrollment is feasible in this group. Additionally, in our cohort, intensive induction therapies lead to superior survival outcomes attributed to successful salvage therapy. These data suggest that strategic therapy sequencing and salvage therapy may be important in optimizing outcomes for *TP53*m AML patients.

## 1. Introduction

Mutations in *TP53* are found in an estimated 5–15% of de novo acute myeloid leukemia (AML). The frequency increases to 20–40% in older patients or those with secondary (s-AML) or therapy-related AML (t-AML) [1]. The presence of a pathogenic *TP53* mutation at a variant allele frequency (VAF) of ≥10% defines AML with mutated *TP53* (*TP53*m AML), a subgroup classified as adverse risk based on the European LeukemiaNet (ELN)’s classification [2]. In addition, biallelic *TP53* alterations, defined as multiple mutations or mutation with concurrent deletion of the other allele, have a major influence on classification of myelodysplastic syndrome in the 5th edition of the World Health Organization (WHO) classification of hematolymphoid tumors and International Consensus Classification (ICC) of Myeloid Neoplasms, but the allelic state of *TP53* does not impact the classification of AML [3,4]. *TP53*m AML represents a distinct disease entity with unique molecular and clinical characteristics. The disease is commonly associated with complex karyotypes, and, in about half of cases, occurs in the absence of other AML-associated gene mutations [5]. Clinically, *TP53*m alterations define the most treatment-resistant AML subtype with poor response to treatment, high relapse rates, and dismal prognosis with 2-year overall survival (OS) under 10% [6,7].

Recent advances have provided a variety of options for up-front AML therapy. The most commonly used regimens are cytarabine with daunorubicin or idarubicin (7 + 3) [8], liposomal cytarabine with daunorubicin (CPX-351) [9,10], and venetoclax combined with a hypomethylating agent (VEN-HMA) or low-dose cytarabine [11,12,13,14]. Other regimens that are used include mitoxantrone with etoposide and infusional cytarabine (MEC) [15], fludarabine with cytarabine, G-CSF, and idarubicin (FLAG-IDA) with or without venetoclax [16,17], single-agent hypomethylating agent (HMA, decitabine or azacitidine), and clofarabine with low-dose subcutaneous cytarabine (Clof-LDAC) [18].

The inferior response of *TP53*m AML to therapy has been documented for all therapy approaches [12,19,20], and the optimal approach for treating these patients has not been well established [21,22]. Since these patients frequently fail to achieve complete remissions (CR) after first-line therapy, salvage therapy is commonly offered. In this study, we performed a retrospective analysis to compare clinical outcomes and the impact of therapy sequence in *TP53*m AML patients treated at our center. The main objectives were to understand the clinical features, treatment patterns, and clinical outcomes of *TP53*m AML to provide guidance for treatment selection and sequencing.

## 2. Methods

### 2.1. Study Design, Patients, and Treatment Characteristics

This retrospective cohort study analyzed data from the AML registry of the University of Minnesota. This study included adult patients (≥18 years old) with a diagnosis of AML or myeloid sarcoma between the dates of 1 January 2014 and 31 July 2022, whose disease was characterized by a next-generation sequencing (NGS) panel within 30 days of the diagnosis. Patients with acute promyelocytic leukemia were excluded from this study. Patients who received any investigational first-line therapy or patients who were treated with supportive care only were excluded from survival analysis. This study was conducted according to the guidelines of the Declaration of Helsinki, approved by the Institutional Review Board of the University of Minnesota, and exempt from patient consent.

Patients with a pathogenic *TP53* mutation by NGS or with loss of the *TP53* gene locus (per chromosome 17p deletion demonstrated by cytogenetic testing) were classified as *TP53*m AML. Patients without a pathogenic *TP53* mutation or 17p deletion were classified as *TP53* wild type (*TP53*wt). Biallelic *TP53* alterations were defined as two *TP53* mutations, or a single *TP53* mutation with VAF > 0.6, or a *TP53* mutation and a 17p deletion by cytogenetics. Normalized biallelic *TP53* alterations were defined as *TP53* mutation VAF that exceeded 0.6 when normalized for bone marrow blast percentage (*TP53* mutation VAF)/(BM blast %).

Treatment was classified as intensive and non-intensive. Intensive treatments were infusional cytarabine with daunorubicin or idarubicin (7 + 3), liposomal cytarabine with daunorubicin (CPX-351), mitoxantrone with etoposide and infusional cytarabine (MEC), and fludarabine with cytarabine, G-CSF, and idarubicin (FLAG-IDA) with or without venetoclax (VEN). Non-intensive treatments consisted of a hypomethylating agent (HMA, decitabine or azacitidine) with or without VEN (VEN-HMA or HMA) or clofarabine with low-dose subcutaneous cytarabine (Clof-LDAC).

### 2.2. Outcomes and Statistical Analyses

Demographic and disease characteristics were obtained from the University of Minnesota AML registry, Bone Marrow Transplant database, and electronic medical records. Descriptive statistics, including frequency, proportion, mean, median, and range, were employed to characterize demographic and clinical outcome variables. Non-continuous variables were described using frequencies and proportions, while continuous variables were described using medians and percentiles. Achievement of complete remission (CR) was based on physician assessment and ELN 2022 response criteria definitions and included CR with partial (CRh) and incomplete hematological recovery (CRi) [2]. Statistical comparison of categorical variables was performed using the chi-squared test. The Kruskal–Wallis test and Wilcoxon rank-sum test were used for comparison of continuous variables between groups. The log-rank test was used for univariate comparisons. Multivariate analysis using Cox proportional hazard models was used to evaluate the impact of clinical characteristics on regimen choice and survival. Kaplan–Meier analysis was used to estimate the probabilities of overall survival (OS), defined as the date of first-line treatment initiation to the date of death from any cause or last follow-up [23]. For patients who were alive at the time of analysis, survival was censored based on the date of analysis, and OS was calculated from the treatment start date to the date of data analysis as opposed to the date of death. All statistical analyses were implemented using SAS version 9.4 (SAS Institute, Cary, NC, USA) and R version 4.20.23 (R Foundation for Statistical Computing, Vienna, Austria). A *p*-value of ≤0.05 was deemed statistically significant.

## 3. Results

### 3.1. Demographic, Disease, and Clinical Characteristics

A total of 423 patients were included in this study. Demographic, disease, and clinical characteristics are summarized in Table 1. Our cohort consisted of 86 patients (20.3%) with *TP53*m AML and 337 patients (79.7%) with *TP53*wt. The median age at diagnosis was 65.3 years for the full cohort and was significantly higher in the *TP53*m group (median 67.3 years versus 64.4 years; *p* = 0.01). The proportion of t-AML and s-AML was significantly higher in the *TP53*m group compared to *TP53*wt (t-AML 22.1% of *TP53*m versus 6.8% of *TP53*wt patients; *p* < 0.01, s-AML 51.2% of *TP53*m versus 29.1% of *TP53*wt patients; *p* < 0.01). The majority of patients with *TP53*m AML (47 of the 70 *TP53m* patients with available VAF data, 67.1%) had *TP53*m VAF of >0.4 (40%). The remainder (23 of the 70 *TP53m* patients with available VAF data, 32.9%) had *TP53*m VAF of 0.1 to 0.4 (10–40%). We identified 96 unique *TP53* mutations in 86 patients. Consistent with prior reports [24], 70 of the 96 (73%) *TP53* mutations in our cohort were missense mutations in the DNA-binding domain (Table 1, Supplementary Tables S1 and S2). Evidence of biallelic *TP53* alterations was found in 56 of the 69 patients with an evaluable allelic state (81.2%). When normalized for bone marrow blast percentage, 65 of these 69 evaluable patients (94.2%) showed evidence of biallelic *TP53* alterations, as seen in other studies [25]. Adverse risk cytogenetics were prevalent in *TP53*m group (57 of the 84 *TP53m* patients with available cytogenetic data, 67.9%). Adverse cytogenetics in this group consisted of complex karyotype (56/84 patients, 66.7%), monosomal karyotype (43/84 patients, 51.2%), −5 or del(5q) (33/84 patients, 39.3%), −7 (22/84 patients, 26.2%), and −17 or abnormal(17p) (20/84 patients, 23.8%). Co-occurrence with other AML-associated mutations such as *FLT3*, *IDH1*, *IDH2*, *NPM1*, *RUNX1*, and *ASXL1* was less common in *TP53*m AML compared to *TP53*wt.

Notably, we observed a significantly lower baseline bone marrow (BM) blast percentage at diagnosis in the *TP53*m group compared to *TP53*wt (median BM blast 33% versus 56%; *p* < 0.01) and a trend toward a lower peripheral blast percentage and blast count (median peripheral blast percentage 9% versus 15.2%; *p* = 0.051; and median peripheral blood blast counts 0.2 × 10^9^ cells/L versus 0.7 × 10^9^ cells/L; *p* = 0.08), consistent with the findings from our prior analysis of the Cancer Genome Atlas (TCGA) and Beat AML datasets [26] and other reports [24,27]. No significant differences in other cell counts (hemoglobin, white blood cells, and platelet counts) and rate of disseminated intravascular coagulation (DIC) or tumor lysis syndrome (TLS) were observed between the two groups. Hyperleukocytosis at presentation was rare in both groups (1.8% of the *TP53*wt cohort and none in the *TP53*m group; *p* = 0.28).

### 3.2. Treatment Patterns, Outcomes, and Survival of TP53m AML

Of the 86 patients with *TP53*m, 66 patients were included in the survival analyses. The most common reasons for exclusion were insufficient/incomplete data primarily due to up-front treatments at other facilities (n = 10). The remainder of excluded patients were those treated with supportive care only (without anti-cancer therapy, n = 8) or enrolled in clinical trials in the front-line setting (n = 2).

**Figure 1:**
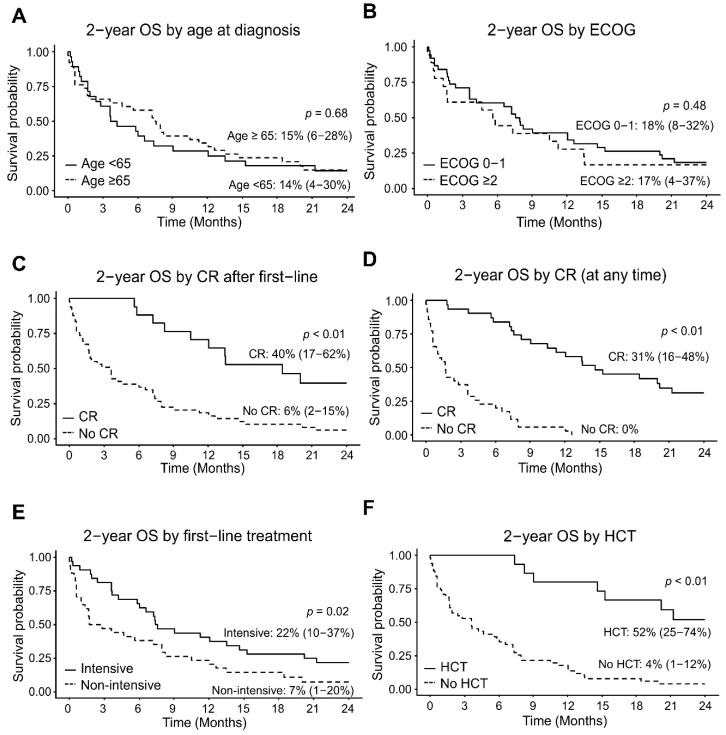
Overall survival by (**A**) age at diagnosis; (**B**) ECOG score; (**C**) CR status after first-line treatment; (**D**) CR status at any line of treatment; (**E**) first-line treatment group (intensive versus non-intensive); and (**F**) treatment with allogeneic HCT.

Of the 66 patients included in the analysis, 32 patients (48.5%) received intensive first-line treatment and 34 patients (51.5%) received non-intensive first-line treatment. A higher proportion of patients aged <65 received intensive first-line treatment (19/28, 67.9%). In contrast, only 13/38 (34.2%) of patients aged ≥65 received first-line intensive treatment (*p* = 0.02). Similarly, patients with a baseline Eastern Cooperative Oncology Group (ECOG) performance status (PS) score of 0–1 received intensive first-line treatment more often than patients with a PS of ≥2, but this trend was not statistically significance (23/38 (60.5%) versus 6/18 (33.3%), respectively, *p* = 0.057). We observed an overall poor prognosis across the cohort of *TP53*m AML with a median OS of 6.4 months and 2-year OS of 15.1%. Among patients treated with supportive care only, the median OS was 1 month (range: 0.1–5.5 months). Among treated patients, there were no significant differences in OS by age group or ECOG PS (Figure 1A,B). Median OS was 4 months for patients aged <65 and 7.5 months for patients aged ≥65 with a 2-year OS of 14% and 15%, respectively (*p* = 0.68). Median OS was 7.9 months for patients with ECOG PS 0–1 and 5.8 months for patients with ECOG PS ≥ 2 with a 2-year OS of 18% and 17%, respectively (*p* = 0.48).

Achieving CR after first-line treatment or at any point was a strong predictive factor of better survival outcomes. The 2-year OS was 40% in patients who achieved CR after first-line treatment versus 6% for those who did not (*p* < 0.01). The 2-year OS of patients who achieved CR at any time was 31% versus 0% for those who did not (*p* < 0.01, Figure 1C,D).

**Figure 2:**
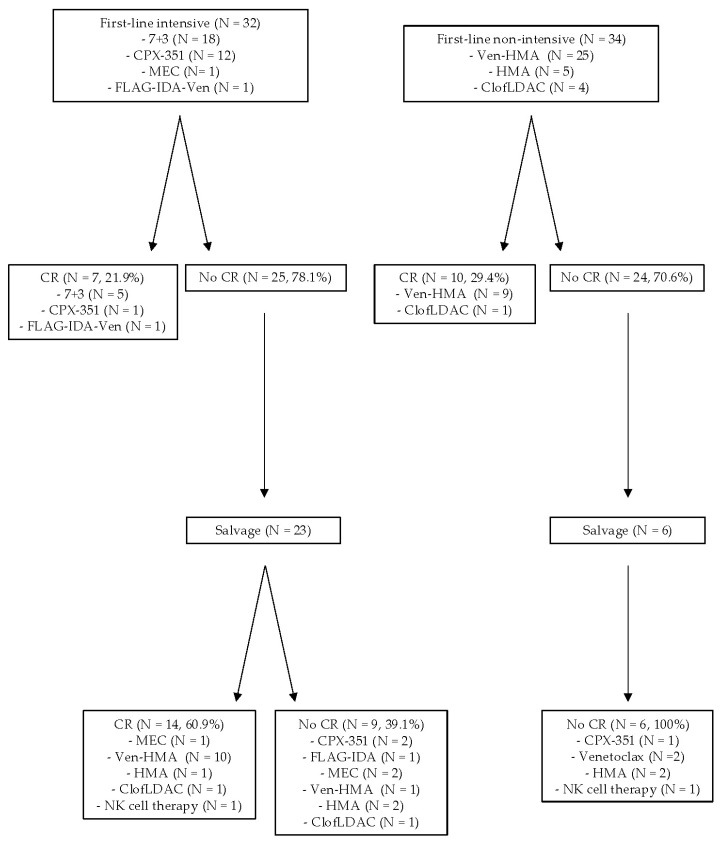
Treatment patterns of *TP53*m AML and CR rates.

We investigated which treatment regimens lead to superior CR rates (Figure 2). Within the intensive first-line group, 7/32 patients (21.9%) attained CR after front-line treatment: 5/18 after 7 + 3, 1/12 after CPX-351, and 1/1 after FLAG-IDA-VEN. Among the 25 patients who did not achieve CR with intensive first-line treatment, 23 patients received salvage treatment, leading to CR in 14/23 (60.9%) of these patients in the salvage setting. Among these patients receiving salvage therapy after intensive induction therapy, 1/6 who received an intensive salvage regimen achieved CR (this patient received MEC), while 12/16 of the patients who received a non-intensive salvage regimen after intensive therapy achieved CR (10/11 patients attained CR after VEN-HMA, 1/3 after HMA single-agent, and 1/2 after Clof-LDAC). Lastly, one of these patients attained CR after investigational allogeneic NK cell therapy as a third-line treatment. With these effective salvage approaches, the cumulative CR rate in the intensive first-line treatment group was 65.6% (21/32 patients).

Within the non-intensive first-line group, 10/34 patients (29.4%) attained CR after front-line treatment (9/25 after VEN-HMA, 1/4 after Clof-LDAC, and 0/5 with HMA monotherapy). Of the 24 patients who did not achieve CR with non-intensive first-line treatment, only 6 patients received salvage treatments: 1 patient received salvage intensive treatment with CPX-351, 4 received single-agent non-intensive treatment (2 VEN and 2 HMA), and 1 received an investigational allogeneic NK-cell therapy. None of the patients in this group (0/6) ever attained CR. The cumulative CR rate in the non-intensive first-line treatment group was 29.4% (10/34). There was no difference in the duration of CR achieved at any line of treatment regarding intensive treatment versus non-intensive treatment (median duration of CR 224 days versus 185 days, respectively, *p* = 0.62). Notably, patients treated with intensive induction therapy who received VEN had a CR rate of 91.6% (11/12 of these patients achieved CR; 11 of these patients received VEN in the salvage setting; and 1 patient received VEN as part of up-front FLAG-IDA-VEN). In contrast, patients treated with non-intensive induction therapy who received VEN had a CR rate of 33% (9/27 patients, *p* = 0.00077; 25 of these patients received VEN up front).

Patients who received intensive treatment as the initial AML-directed therapy demonstrated superior 2-year OS compared to patients receiving non-intensive first-line therapy (median OS 7.6 months versus 2.3 months, 2-year OS 22% vs. 7%; *p* = 0.02, Figure 1E). Among the intensive first-line treatment cohort, the 18 patients who received 7 + 3 demonstrated superior OS in comparison to the 12 patients receiving CPX-351 (median OS 11.2 months versus 7.4 months and 2-year OS 22% versus 0%; *p* = 0.02). As expected, a high proportion of patients receiving CPX-351 had s-AML or t-AML (9/18 (50%) of 7 + 3 patients and 8/12 (67%) of CPX-351 patients had s-AML or t-AML). Patients with *TP53*m s-AML or t-AML had worse survival outcomes compared to de novo *TP53*m AML (median OS 4.2 months versus 8.2 months, 2-year OS 10.5% versus 21.4%, respectively). The proportion of patients with complex cytogenetics was comparable between the 7 + 3 group and CPX-351 (10/18 (55.6%) of the 7 + 3 patients versus 7/12 (58.3%) of the CPX-351 patients). We observed inferior OS in patients with *TP53*m AML with complex cytogenetics versus *TP53*m AML without complex cytogenetics (median OS 5.2 months versus 13.5 months, 2-year OS 8.7% versus 30%, respectively).

The percentage of patients who underwent treatment with allogeneic hematopoietic cell transplantation (allo-HCT) in the intensive first-line group was 37.5% (12/32), which is significantly higher than with the non-intensive first-line group (8.8% (3/34), *p* = 0.01). Patients who received treatment with allo-HCT demonstrated better 2-year OS compared to patients who did not receive allo-HCT (median OS, not reached versus 3.6 months, 2-year OS 52% versus 4%, respectively; *p* < 0.01, Figure 1F). As baseline clinical characteristics can influence the choice of regimen and potentially contribute to the OS difference observed, multivariate analysis using Cox proportional hazard models was performed. Age at diagnosis, ECOG score, first-line regimen type, and treatment with allo-HCT were factors considered in the analysis. Age at diagnosis, first-line regimen type, and treatment with allo-HCT were significantly correlated. After adjusting for the effects of the ECOG score (Table 2)**,** the trend in the survival difference was not statistically significant between the non-intensive first-line treatment versus the intensive first-line groups (adjusted hazard ratio (HR) 1.76 (95%CI: 0.96–3.23), *p* = 0.07).

## 4. Discussion

*TP53*m defines the most treatment-refractory AML subtype with a dismal prognosis. Strategies for treating *TP53*m AML are highly heterogeneous, and an optimal treatment approach has not been defined. The National Comprehensive Cancer Network recommends intensive induction therapy for all eligible AML patients with favorable or intermediate risk disease [28]. In recognition of the dismal outcomes of *TP53m* AML, non-intensive regimens are listed as acceptable first-line options in fit, induction-eligible patients [11,29]. Thus, we performed a retrospective analysis using the AML registry at our institution to define clinical characteristics, treatment response and sequencing patterns, and outcomes for *TP53*m AML.

Our study is consistent with previous studies in demonstrating the poor OS of *TP53*m AML patients [27,30,31]. Prior reports have focused on the impact of front-line therapy on treatment outcomes in *TP53*m AML. A previous study has demonstrated improved CR rates with VEN and azacitidine compared to azacitidine alone in AML patients with poor-risk cytogenetics and *TP53* mutations, but the value of adding venetoclax has not been confirmed in other studies [32,33]. A recent meta-analysis of treatment outcomes for newly diagnosed, treatment-naïve *TP53*m AML demonstrated similar CR rates for intensive therapy and VEN-HMA (46 and 49%) [19]. Notably, a recent study of 370 newly diagnosed AML patients with *TP53*m or a deletion of 17p revealed a modest difference in OS between patients receiving intensive induction therapy or VEN-HMA (median OS 9.4 versus 7.4 months). However, these differences diminished after adjusting for relevant baseline characteristics and allo-HCT treatment [21]. These studies, like ours, demonstrated significantly worse outcomes for HMA alone. However, another retrospective analysis revealed that OS and event-free survival (EFS) were not significantly different among the induction regimen groups (intensive regimen versus HMA with or without venetoclax), with a modest survival benefit seen in the allo-HCT group [22]. In contrast to our study, these reports did not include an analysis of salvage regimens or regimen sequencing in their analysis.

One of the most important prognostic factors for AML is achieving CR. Since CR rates are low in *TP53*m patients, these patients are often exposed to multiple lines of therapy. However, the value of salvage therapy and optimal sequencing approaches have not been described in this patient population. Due to known poor outcomes with intensive chemotherapy, many providers are favoring less-intense strategies for patients with *TP53*m AML due to less toxicity and high-grade adverse events; however, the efficacy of these approaches have not been compared to intensive options. In our cohort, very few patients treated non-intensively in the first line received salvage therapy. Non-intensive regimens can take several cycles to achieve optimal responses. During this time, patients are often profoundly cytopenic and highly susceptible to complications, which can render them ineligible for alternate therapies. Our observation suggests that starting with intensive induction therapy in eligible patients may yield superior outcomes. These patients can be effectively salvaged with non-intensive treatment, especially HMA with venetoclax. Patients treated intensely in the first line demonstrated superior OS despite having a slightly lower CR rate to first-line therapy, compared to the first-line non-intensive group.

Our data also suggest that, if non-intensive treatment is indicated, HMA with venetoclax is superior to single-agent HMA, despite these *TP53*m patients presenting low response rates to venetoclax combinations compared to *TP53*wt patients. Consistent with other studies, allo-HCT offers major survival benefits for appropriate candidates [21,34]. However, the rate of relapse is high, and survival remains poor even after HCT in *TP53*m AML [35].

As a retrospective study, the observed difference in OS between treatment types may be confounded by age at diagnosis and baseline PS. The absence of OS difference between patients aged <65 and ≥65 at the time of diagnosis and between patients with ECOG 0–1 and ≥2 implies appropriate patient selection in our cohort, highlighting the universally treatment-refractory nature of *TP53*m AML. Patients treated with intensive first-line therapy were generally younger, had a better PS score, a higher proportion of them were eligible for salvage treatment, and they received allo-HCT more frequently. Patients who received non-intensive treatment as their initial therapy had more co-morbidities, a lower baseline PS score, and more advanced age. These factors likely contributed to the shorter OS in the non-intensive first-line group. Since the age at diagnosis, first-line treatment regimen, and allo-HCT were correlated, we only included the first-line regimen in the multivariate analysis model. After making an adjustment using the ECOG score, only a marginal association with superior 2-year OS was observed in the intensive first-line treatment group. No significant difference in the HR was demonstrated between non-intensive first-line treatments versus intensive first-line regimens.

## 5. Conclusions

In summary, this single-center retrospective cohort study shows that *TP53*m AML patients have poor OS with currently available treatments. Allo-HCT is the only therapy associated with long-term disease control. Achieving CR at any point is a strong predictive factor of a better OS. Our observation suggests that eligible patients may benefit from intensive front-line induction therapy and that they can be effectively salvaged with non-intensive regimens, especially HMA with venetoclax, if CR is not achieved after intensive treatment first-line therapy. Indeed, VEN-HMA combinations are being considered for maintenance therapy in AML [36]. Moreover, we revealed unique presenting characteristics of *TP53*m AML (lower BM and peripheral blasts and a lower rate of hyperleukocytosis). These features suggest that *TP53*m AML patients are less likely to present with hematologic emergency conditions, which may give practitioners time to carry out clinical trial evaluations and enrollments.

## Figures and Tables

**Table 1:** Patient demographics and disease characteristics.

	All Groups	*TP53*m	*TP53*wt	*p*-Value
N	423	86 (20.3%)	337 (79.7%)	
Age at diagnosis	65.3 (18.7–92.9)	67.3 (28.9–87.1)	64.4 (18.7–92.9)	0.01
Gender				
Male	252 (59.6%)	57 (66.3%)	195 (57.9%)	0.16
Female	171 (40.4%)	29 (33.7%)	142 (42.1%)	
Therapy-related AML	42 (9.9%)	19 (22.1%)	23 (6.8%)	<0.01
Secondary AML	142 (33.6%)	44 (51.2%)	98 (29.1%)	<0.01
ELN2022 risk category (N = 302)				
Favorable	11 (2.6%)	0	11 (3.3%)	<0.01
Intermediate	59 (14.0%)	0	59 (17.5%)	
Adverse	232 (54.9%)	86 (100%)	146 (43.3%)	
*TP53*m VAF, median (N = 70)		0.65 (0.18–0.94)		NA
0.1–0.4		23 (32.9%)		
>0.4		47 (67.1%)		
*TP53* allelic state (N = 69)				
Biallelic *TP53* alteration *		56 (81.2%)		NA
Biallelic *TP53* alteration (normalized) **		65 (94.2%)		
*TP53* mutations (total = 96)				
DNA-binding domains (DBD)		78 (81.2%)		NA
Missense		70 (72.9%)		
Truncation, Frameshift, Deletion		8 (8.3%)		
Non-DBD (other domains)		18 (18.8%)		
Splice donor/acceptor sites		6 (6.3%)		
Cytogenetics, *TP53*m AML (N = 84)				NA
Normal cytogenetics		16 (19%)		
Complex karyotype		56 (66.7%)		
Monosomal karyotype		43 (51.2%)		
−5 or del(5q)		33 (39.3%)		
−7		22 (26.2%)		
−17 or abn(17p)		20 (23.8%)		
ELN2022 cytogenetic risk category, *TP53*m AML (N = 84)				NA
Favorable		0		
Intermediate		27 (32.1%)		
Adverse		57 (67.9%)		
Co-mutations				
* FLT3*	61 (14.4%)	3 (3.5%)	58 (17.2%)	<0.01
* IDH1/IDH2*	76 (18.0%)	4 (4.7%)	72 (21.4%)	<0.01
* NPM1*	57 (13.5%)	1 (1.2%)	56 (16.6%)	<0.01
* RUNX1*	37 (8.8%)	0 (0%)	37 (11.0%)	0.01
* ASXL1*	102 (24.1%)	14 (16.3%)	88 (26.1%)	<0.01
* KRAS/NRAS*	83 (19.6%)	10 (11.6%)	73 (21.7%)	0.47
* KIT*	13 (3.1%)	1 (1.2%)	12 (3.6%)	0.31
Baseline BM blast (%)	50 (1–98)	33 (1–94)	56 (9–98)	<0.01
Baseline Hgb (g/dL)	8.5 (2.2–14.6)	8.3 (5.0–14.5)	8.6 (2.2–14.6)	0.48
Baseline WBC (10^9^ cells/L)	3.2 (0.1–329.9)	3.1 (0.1–82.8)	3.2 (0.1–329.9)	0.60
Baseline peripheral blast (%)	13.8 (0–100)	9 (0–78)	15.2 (0.7–100)	0.051
Peripheral blast count (10^9^ cells/L)	0.6 (0–300.2)	0.2 (0–31.7)	0.7 (0–300.2)	0.08
Baseline ANC (10^9^ cells/L)	0.2 (0–38.2)	0.4 (0–23.8)	0.5 (0–38.2)	0.41
Baseline platelet count (10^9^ cells/L)	61 (4–1019)	47 (7–1019)	64 (4–867)	0.26
DIC at presentation	18/227 (7.9%)	5/45 (11.1%)	13/182 (7.1%)	0.38
TLS at presentation	11/181 (6%)	2/36 (5.6%)	7/145 (4.8%)	0.25
Hyperleukocytosis at presentation	5/334 (1.5%)	0/63 (0%)	5/271 (1.8%)	0.28

* Biallelic *TP53* alterations were defined as 2 *TP53* mutations, a single *TP53* mutation with VAF > 0.6, or a *TP53* mutation and a 17p deletion by cytogenetics. ** Biallelic *TP53* alterations (normalized) were defined as *TP53* mutation VAF that exceeds 0.6 when normalized for bone marrow blast percentage: Normalized VAF = (*TP53* mutation VAF)/(BM blast %). NA = not applicable.

**Table 2:** Multivariate Cox proportional hazard model for OS.

	Hazard Ratio (95%CI)	*p*-Value
First-line intensive	1.00	0.07
First-line non-intensive	1.76 (0.96–3.23)	
ECOG 0–1	1.00	0.83
ECOG ≥ 2	1.07 (0.57–2.03)	

## Data Availability

The data presented in this study are only available upon request from the corresponding author due to confidentiality and ethical restrictions.

## Supplementary Materials

The following supporting information can be downloaded at: https://www.mdpi.com/article/10.3390/cancers16162784/s1. Table S1: Comparison of Outcomes by TP53 allele status. Table S2: TP53 alteration type and outcomes.
